# Adapting behavioral activation for patients receiving medications for opioid use disorder in primary care: a pilot study

**DOI:** 10.3389/fpsyg.2024.1439946

**Published:** 2024-10-08

**Authors:** Stephanie A. Hooker, Hanmin Kim, Mary Lonergan-Cullum, Andrew M. Busch, Tanner Nissly, Robert Levy

**Affiliations:** ^1^Research and Evaluation Division, HealthPartners Institute, Minneapolis, MN, United States; ^2^Department of Family Medicine and Community Health, University of Minnesota Medical School, Minneapolis, MN, United States; ^3^Hennepin Healthcare, Minneapolis, MN, United States; ^4^Department of Medicine, University of Minnesota Medical School, Minneapolis, MN, United States

**Keywords:** psychotherapy, values, opioids, feasibility, acceptability, behavioral therapy, substance use disorders, buprenorphine

## Abstract

**Introduction:**

Effective adjunctive therapeutic treatments for patients with opioid use disorder (OUD) on medication for OUD (MOUD) in primary care settings are needed to address high rates of mental illness and stress. Behavioral activation (BA) is a brief, evidence-based therapy that has potential to improve quality of life in people with OUD. The purpose of this pilot study was to evaluate the feasibility and acceptability of values-based BA (VBA) as an adjunct treatment for patients receiving MOUD in primary care.

**Methods:**

Participants were recruited for a single-arm pilot trial of BA in a primary care setting. VBA was adapted for people with OUD and included 4–6 sessions delivered over 12 weeks with a behavioral health consultant, either in-person or virtually. Feasibility was assessed as recruitment percent and pace and retention percent. Acceptability was assessed with the Client Satisfaction Questionnaire-8 (CSQ-8). Participants completed self-report measures of well-being, depression, substance use, and psychological processes of change at baseline, mid-intervention (6-weeks), and post-intervention (12-weeks). Participants engaged in a brief interview about their experiences at the end of the intervention.

**Results:**

Twenty-one participants enrolled in the intervention (66.7% female, *M* age = 44.0 years, 19% of those invited). Participants completed an average of 5.1 BA sessions (*SD* = 1.6) and most (90%) were retained through 12 weeks. Participants rated the intervention as highly acceptable on the CSQ-8 (*M* = 30.4/32.0, *SD* = 1.6). In qualitative interviews, participants reported that working with the therapist and setting values-based goals were helpful, while also recommending more tailoring to patients’ needs and offering the program early in MOUD treatment. Preliminary efficacy data suggest the program was associated with small to moderate improvements in life satisfaction (Cohen’s *d* = 0.25) and positive affect (*d* = 0.62), whereas there were no changes in depression (*d* = 0.09) or negative affect (*d* = −0.07) in a group with low depression at baseline.

**Discussion:**

VBA adapted for patients on MOUD in primary care was feasible to deliver and acceptable to participants. Minor modifications to the target population and treatment manual could increase the program’s impact. Future studies will test the efficacy of the intervention in improving quality of life and OUD treatment outcomes.

**Clinical trial registration:**

https://clinicaltrials.gov/study/NCT05262725, Unique ID: NCT05262725.

## 1 Introduction

Opioid misuse is a significant concern in the United States, with over 75% of the 106,699 drug overdose deaths in 2021 involving an opioid, and 88% of opioid-involved deaths linked to synthetic opioids (Centers for Disease Control and Prevention, 2023). Medications for opioid use disorder (MOUD), such as buprenorphine, are useful in managing opioid use disorder (OUD) and reducing the risk for overdose and deaths by 48–62% (Samples et al., 2023; Sun et al., 2022).

Although MOUD help prevent premature mortality due to overdose, these medications alone do not significantly improve mental health and well-being for persons with OUD (Hooker et al., 2021). Approximately half of patients who initiate MOUD treatment in primary care have mental health symptoms, including anxiety, depression, and posttraumatic stress disorder (Haider et al., 2020; Hooker et al., 2020). In addition, many patients also experience significant psychosocial concerns, notably unemployment, lower educational attainment, criminal justice involvement, low income, food insecurity, and unreliable transportation (Haider et al., 2020; Hooker et al., 2020). MOUD are effective in reducing drug cravings and preventing overdoses, however, evidence suggests that MOUD may reduce depression and anxiety symptoms in the short-term (1 month after treatment initiation), but these reductions may not be sustained at 6 months (Hooker et al., 2021; Na et al., 2022).

Psychotherapy research investigating behavioral and cognitive strategies to enhance OUD treatment is limited, and available data from the research literature are mixed on the efficacy of psychotherapeutic strategies for improving outcomes in persons with substance use disorders (Carroll and Weiss, 2017; Fiellin et al., 2013; Martin et al., 2019; Moore et al., 2012; Neumann et al., 2013). Several behavioral approaches have been used to address comorbid mental health concerns in MOUD programs with disappointing findings. Specifically, one systematic review found that behavioral treatment did not add any benefit across a variety of outcomes, including opioid abstinence (Amato et al., 2011). Another review indicated that half the identified behavioral treatments did not yield additional benefits to people on MOUD maintenance therapy, although there may be some benefit to using contingency management (Carroll and Weiss, 2017). However, the authors noted that the nature of the interventions that have been studied have been broad (e.g., cognitive-behavioral therapy, contingency management, traditional drug counseling), the interventions have mixed effects on different outcomes (i.e., retention v. abstinence), and it is not clear who may benefit from different types of therapy (Carroll and Weiss, 2017). This suggests that further work is needed to identify adjunctive treatments that may be beneficial for people with OUD on MOUD.

Recovery from a substance use disorder, like OUD, is a unique experience that might contribute to or exacerbate existing mental health challenges. Reinforcement theory suggests that substance use disorders are developed and maintained because of a lack of positive reinforcement in the environment from non-substance use-related activities (Rogers et al., 2008; Vuchinich and Tucker, 1988). The substance then provides reinforcement that is lacking from other areas. Then, in recovery, individuals often have to withdraw from activities that used to bring them pleasure - not only using the substance they are trying to avoid, but also the social environments and contacts that may have been associated with substance use. This could lead to social isolation or loneliness that puts patients at risk for relapse (Polenick et al., 2019). Moreover, prior to entering recovery, some people with substance use disorders spend a lot of time using substances or engaging in activities to obtain substances (American Psychiatric Association, 2013). Indeed, evidence suggests that college students who engage in heavy alcohol use engage in fewer substance-free positive activities and report less pleasure from those activities than peers who do not use alcohol heavily (Correia et al., 2003). Without those routines, patients may be left without non-drug related positive environmental reinforcement and clear goals or directions (Magidson et al., 2011). In addition to the narrowed behavioral repertoire seen in substance use disorders, people with substance use disorders also experience greater anhedonia, or the impaired capacity to experience pleasure (Garfield et al., 2014). Withdrawal from opioids and replacement with a partial opioid agonist, like buprenorphine, may reduce patients’ capacity to naturally derive pleasure from their activities and environments. However, evidence suggests that people with OUD on opioid agonist medication still experience mood brightening in response to non-drug rewards (Stull et al., 2021). Thus, treatments to improve mood and wellbeing in the context of OUD treatment recovery should guide patients to find positive, non-drug, rewarding activities that bring direction and purpose to patients’ lives and encourage positive social interactions.

One treatment that addresses these deficits is behavioral activation (BA), which is a behavioral treatment that helps patients engage in activities that break the cycle of avoidance, inactivity, and social withdrawal (Jacobson et al., 2001; Kanter et al., 2009). In this capacity, BA can help broaden patients’ behavioral repertoires, or the number and types of non-drug positive activities that they can do, while also providing graded pleasurable activities that provide positive reinforcement and reward. BA has been shown to be as effective as cognitive-behavioral therapy for treating depression, and costs much less to administer (Richards et al., 2016). Given the success of BA for treating depression, adaptations of BA for other conditions (e.g., acute coronary syndrome, chronic pain, diabetes, smoking cessation) are emerging (Busch et al., 2017; Gathright et al., 2022; Hooker et al., 2019; Kim et al., 2017; MacPherson et al., 2010; Vickery et al., 2023). Additionally, others have used BA to treat substance use disorders in residential treatment settings, for patients on methadone maintenance treatment, and for patients with co-occurring substance use and HIV (Anvari et al., 2023; Daughters et al., 2018; Daughters et al., 2016; Magidson et al., 2021; Magidson et al., 2011; Magidson et al., 2022). More recently, others have integrated group-based BA into treatment for pregnant people with OUD in a substance use treatment program embedded in a maternal fetal medicine clinic (Vilensky et al., 2024). BA can serve as a stand-alone treatment (Jacobson et al., 2001) or as a part of a multi-component treatment, such as cognitive behavioral therapy or acceptance and commitment therapy (ACT; Okifuji and Turk, 2015).

Traditionally, BA has encouraged patients to engage in activities that are pleasurable or encourage mastery (Dimidjian et al., 2011). However, BA interventions addressing values may augment motivation and sustain engagement in the behavior (Kanter et al., 2010). This mechanism of action is emphasized in ACT, a cognitive and behavioral therapy that uses values-based action or committed action, as a key component (Hughes et al., 2017). Specifically, ACT helps patients clarify their values and plan values-consistent activities (also known as committed action), which is similar to BA in that it encourages patients to engage in activities that are valuable and meaningful to them. In recovery from OUD, patients may choose to set goals or engage in activities they have avoided because of their OUD.

Therefore, to enhance motivation and encourage patients to reconnect with activities that are valuable and meaningful to them, we chose to adapt the traditional BA treatment with an added focus on values. Values-based BA (VBA) is a promising intervention for patients with OUD receiving treatment in primary care, as VBA is relatively straightforward, time-efficient, and does not require complex skills for the patient. Initially, patients complete a values assessment in which they identify personal values in various life domains. With the assistance of a behavioral health clinician (BHC), patients set incremental behavior goals to move their daily actions to better align with their self-reported values. Goal completion allows the patient to achieve outcomes consistent with recovery, and they potentially experience positive reward, enjoyment, self-efficacy, and confidence as they progress through the treatment.

To our knowledge, there have been no studies examining VBA as an adjunct treatment for people with OUD receiving MOUD in primary care settings. Thus, the purpose of this pilot study was to deliver VBA as an adjunctive treatment for people with OUD receiving MOUD in primary care, with a specific goal to understand the feasibility and acceptability of the intervention and study design (i.e., recruitment strategies, outcome assessments). In addition, we gathered data on preliminary efficacy of the treatment to improve well-being and mental health, and whether the intervention was associated with increases in the targeted psychological processes of the intervention effect.

## 2 Materials and methods

### 2.1 Study design and setting

The study used a single-arm open pilot trial design conducted at a family medicine residency primary care clinic located in Minneapolis, MN. The study protocol and materials were reviewed and approved by the Institutional Review Board at the University of Minnesota (STUDY00013874). This study was also preregistered on ClinicalTrials.gov (NCT05262725).

### 2.2 Participants

**Figure 1:**
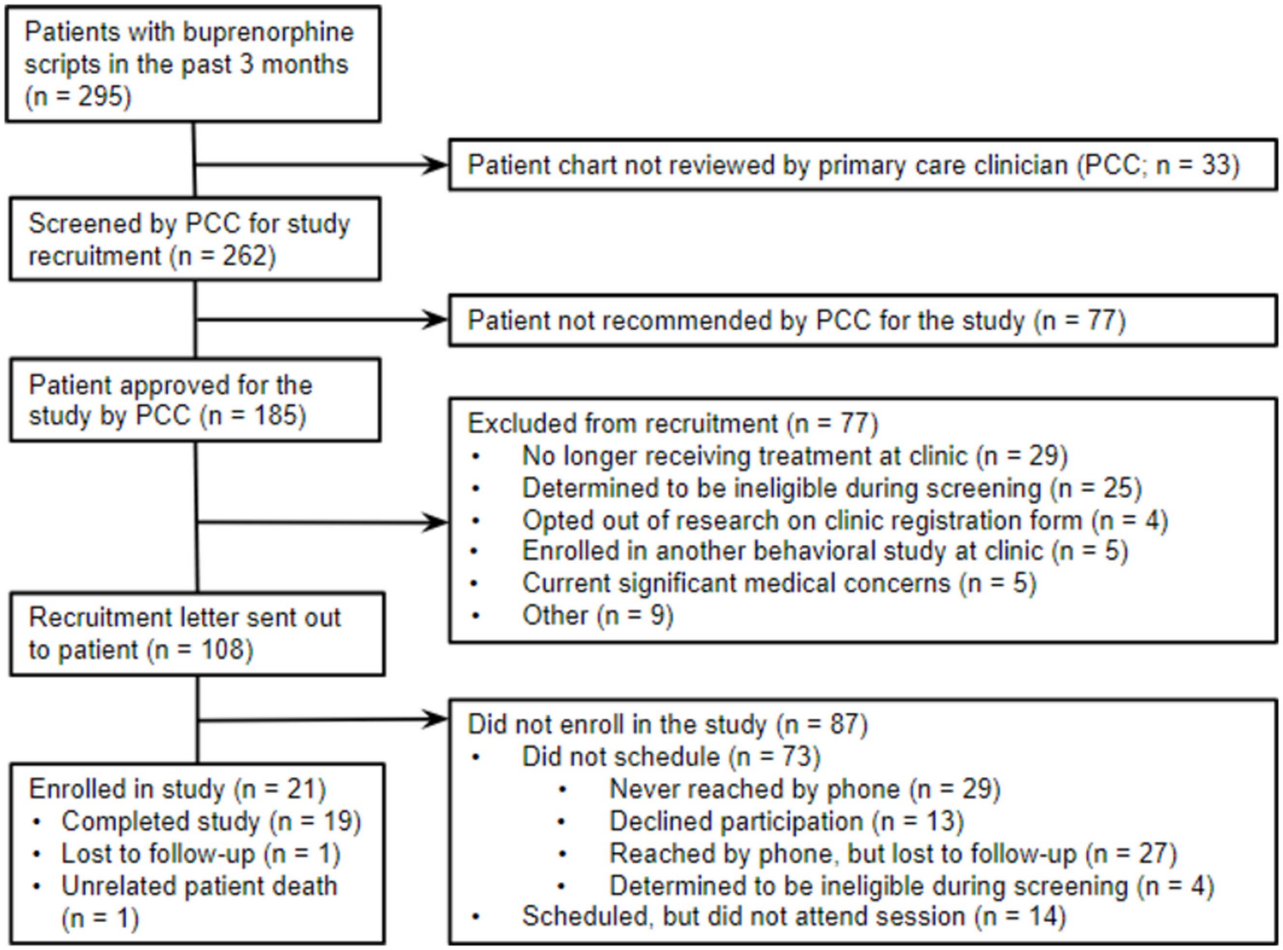
CONSORT study flow diagram.

Potentially eligible participants were identified through reporting and chart review of electronic health records (EHR) (see Figure 1 for CONSORT diagram). The inclusion criteria for the study were (1) current patients at the clinic; (2) at least 18 years of age; (3) diagnosed with OUD; and (4) having an active prescription for buprenorphine or buprenorphine-naloxone for a minimum of one month. Individuals who received concurrent individual psychotherapy with a psychologist or counselor at least monthly were excluded from the study. Patients with active suicidal ideation, intention, or a plan within the past 30 days were deemed ineligible due to their need for a higher level of care. Additional exclusion criteria included dementia, developmental disabilities, or cognitive functioning too impaired to participate in therapy. The research team consulted with patients’ primary care clinicians (PCCs) on these eligibility criteria prior to recruiting any patient. Patients were instructed that their participation was completely voluntary, and their treatment at the clinic would not be affected if they chose not to participate in the study. For additional safeguards for this population, we obtained a certificate of confidentiality through the Substance Abuse and Mental Health Services Administration (SAMHSA) for this study. This certificate of confidentiality protected participants’ privacy by prohibiting disclosure of identifiable, sensitive research information (e.g., substance use behavior) without their consent. After confirming eligibility, 21 patients provided informed consent to participate in this study.

### 2.3 Intervention description

The study behavioral health clinician (BHC) was a licensed master’s level social worker (MSW) who had general psychotherapy training but not BA-specific training prior to training in the study intervention. After receiving focused training, the BHC conducted 4–6 sessions of VBA with participants over the course of 12 weeks, with one session scheduled every 2–3 weeks. Patients had the option to conduct the VBA sessions with a clinician either in-person at the clinic or through virtual telehealth appointments (i.e., videoconferencing or telephone visit). The first session was linked to an in-person clinic encounter, during which patients completed all baseline measures and the first VBA counseling session. The first session lasted up to an hour, and follow-up appointments were 30-min. The BHC followed a detailed intervention manual.

*Session 1*: During the first VBA session, patients met with the BHC and received an introduction to VBA, including the rationale for the intervention and how it complements OUD treatments at the clinic. Next, each patient completed a values assessment centering values on recovery and avoiding opioids. The BHC and the patients discussed what patients valued in life before opioid misuse and how opioids made it challenging to align their behavior with their values. Patients were asked to consider how staying sober would allow them to participate in activities consistent with their values and if sobriety might make participation in some activities challenging. After reviewing values, patients set two or three activation goals. One goal targeted OUD treatment and explicitly supported them staying abstinent from substances, such as attending MOUD appointments, taking MOUD, seeking out non-drug friendships and social support, or attending support groups. The second goal was a values-congruent goal that could also be pleasurable. For example, if a patient identified “Family” as an important value domain, they might set a goal to connect with a family member for conversation, dinner, or other shared activity in the next two weeks. If patients felt that this second goal was not something inherently fun or pleasurable, then they set a third goal specifically to engage in an enjoyable activity, such as reading a book, taking a warm bath, going to a movie, or taking a walk in nature. After goal setting, the clinician wrapped-up the session and scheduled the follow-up appointment.

*Sessions 2–6*: Follow-up sessions occurred every 2–3 weeks. At the start of each session, the BHC engaged in rapport building and agenda setting. After informally assessing patients’ mood, the BHC asked patients to reflect on progress made on goals since the previous session. First, the BHC assessed treatment engagement; specifically, whether the patient attended any scheduled office-based opioid treatment (OBOT) appointments, took their MOUD, abstained from substances, and attended support groups. Progress and struggles on this goal and other VBA goals were addressed. The BHC briefly reminded the patient of their values and helped connect these values to their goals set for the next few weeks. Next, the BHC helped patients set new VBA goals, which could be either to continue previous goals, revise previous goals, or set new goals in a different domain if the original goals were no longer relevant. The objective was to grade the goals over the course of treatment, so that incremental steps could be taken to reach larger, longer-term goals. Once review of progress and goal setting were completed, the BHC wrapped-up the session and scheduled a follow-up visit. At the final session, the BHC worked with the patient to identify ways they could continue to move forward on these goals. Both short-term and long-term goals were discussed.

### 2.4 Measures

#### 2.4.1 Demographic and clinical characteristics

Participants self-reported age, gender, race, ethnicity, employment status, educational attainment, income level, and marital status. Participants also self-reported the number of months they had been taking buprenorphine.

#### 2.4.2 Substance use history

Items D1-D13 of the Alcohol/Drugs section of the Addiction Severity Index (ASI), 5th edition, were used to assess substance use (McLellan et al., 1992a, 1992b). Participants reported the number of days in the past 30 days they used the following substances: alcohol (any use), alcohol (to intoxication), heroin, methadone, other opiates/analgesics, barbiturates, sedatives/hypnotics/tranquilizers, cocaine/crack, methamphetamines or other amphetamines, cannabis/marijuana, hallucinogens/psychedelics, inhalants, and more than one substance per day (including alcohol). Participants were asked to report the route of administration (i.e., oral, nasal, smoking, intravenous injection [IV], non-IV injection) for any substance they reported using in the past 30 days. The ASI has demonstrated test–retest reliability, as well as concurrent, discriminant, and predictive validity, making it a useful assessment for clinical and research purposes (McLellan et al., 1985).

#### 2.4.3 Feasibility

Intervention feasibility was evaluated with three key elements: recruitment percent, recruitment pace, and retention percent (Pfledderer et al., 2024). Recruitment percent was the percentage of patients invited to participate who enrolled in the intervention. Recruitment pace was the number of patients recruited every week and the time required to achieve enrollment targets. Retention percentage was the percentage of participants who completed all VBA sessions among those who enrolled in the study. Recruitment metrics were chosen to determine the proportion of patients who would agree to participate and rate of recruitment into the trial, both of which could be used for planning a future larger trial. Retention percentage was used to determine whether participants would complete the planned treatment sessions and whether adjustments would be needed in the future.

#### 2.4.4 Acceptability

The 8-item Client Satisfaction Questionnaire (CSQ-8; Larsen et al., 1979) was used to assess treatment acceptability. Participants rated the quality of the service and their satisfaction with the services provided on a 4-point Likert-type scale with varying response options (e.g., “How would you rate the quality of the service you received? 4 = Excellent, 3 = Good, 2 = Fair, or 1 = Poor). Items were summed for a total score ranging from 8 to 32, with higher scores corresponding to greater satisfaction with treatment.

#### 2.4.5 Clinical outcome

As a secondary clinical outcome, we assessed opioid abstinence. Urine toxicology (U-Tox) results were collected at baseline, mid-treatment, and post-treatment as part of the research study (not usual care). The rapid urine drug screening panel used for the study tests for marijuana, phencyclidine (PCP), cocaine, methamphetamine, morphine, amphetamine, benzodiazepine, tricyclic antidepressants (TCA), methadone, barbiturate, oxycodone, propoxyphene (PPX), and buprenorphine. For this study, we report the prevalence of positive opioid screens (a sign of potential relapse) and negative buprenorphine screens (a measure of adherence to treatment) at each time point.

#### 2.4.6 Psychological outcomes

Changes in depression, affect, and life satisfaction were assessed as secondary psychological outcomes. Patients completed the Patient Health Questionnaire (PHQ-9; Kroenke and Spitzer, 2002) at baseline to screen for depression. This 9-item questionnaire uses a Likert-type scale by which respondents indicate if they have experienced a symptom, such as “Feeling down, depressed, or hopeless,” in the past week 0 = Not at all, 1 = Several days, 2 = More than half the days, or 3 = Nearly every day. Summed items scores equal to or greater than 5, 10, 15, or 20 indicate mild, moderate, moderately severe, and severe depression severity, respectively.

The Center of Epidemiologic Studies Depression Scale (CES-D-10; Andresen et al., 1994) was completed by patients at baseline, mid-treatment, and post-treatment to track changes in depressive symptoms following the BA intervention. Similar to the PHQ-9, patients were asked to report how often they felt during the past week to statements, such as “I was bothered by things that usually do not bother me” or “I felt depressed,” with 0 = Rarely or none of the time (less than 1 day), 1 = Some or a little of the time (1–2 days), 2 = Occasionally or a moderate amount of time (3–4 days) or 3 = All of the time (5–7 days). Of the 10 items, statements 5 and 8 were reversed scored. Higher composite scores on the CES-D-10 indicate greater levels of depressive symptoms. Internal consistency of the CES-D was acceptable at baseline (Cronbach’s *α* = 0.70) and high at 6- and 12-weeks (α = 0.83 and 0.87, respectively).

Patient mood was assessed at baseline, mid-treatment, and post-treatment with a positive and negative mood scale previously used to record daily mood (Hooker and Masters, 2018; Steger et al., 2008). Positive affect terms included relaxed, proud, excited, appreciative, enthusiastic, happy, satisfied, and curious. Five items - sluggish, afraid, sad, anxious, angry - were utilized to assess negative affect. Items were selected from the Positive and Negative Affect Scale (PANAS; Watson et al., 1988), but more common mood items, such as happy, sad, and angry were added. Participants reported to what extent they felt that way during the past week, with Likert-type options of 1 = Very slightly or not at all, 2 = A little, 3 = Moderately, 4 = Quite a bit, 5 = Extremely. Internal consistency was acceptable for both the positive affect (*α*s = 0.74–0.86) and negative affect scales (αs = 0.67–0.84) across all three time points.

The Satisfaction with Life Scale (SWLS; Diener et al., 1985) was administered at baseline and post-treatment to measure changes in life satisfaction across the study. Participants indicated their current level of agreement (7 = Strongly agree) or disagreement (1 = Strongly disagree) with the 5 statements, such as “In most ways my life is close to ideal.” Items are summed for a total score, with higher scores corresponding to greater life satisfaction. Scores below 20 indicate some degree of dissatisfaction, whereas scores above 20 indicate higher levels of life satisfaction. The SWLS was highly internally consistent at both time points (*α* =0.86 and 0.92 at baseline and post-intervention, respectively).

#### 2.4.7 Intervention processes

We collected measures to assess the impact of the intervention on hypothesized processes at baseline, mid-treatment, and post-treatment. We measured the patients’ changes in activation using the Behavioral Activation for Depression Scale (BADS 9-item; Manos et al., 2011). For each of the statements, participants were asked to select the response that best describes how much the statement was true for them during the past week, from 0 = Not at all to 6 = Completely. Example statements include “I engaged in many different activities” and “I did things that were enjoyable,” as well as reversed scored items “There were certain things I needed to do that I did not do.” Two subscales from the BADS were summed for a total score: Activation (engagement in pleasurable activities) and Avoidance (refraining from pleasurable activities, reverse scored). Higher scores on the combined scale indicate greater activation.

Valued directions were assessed with eight items from the Valued Action Subscale of the CompACT (Francis et al., 2016). For each statement, participants rated their level agreement from 0 = Strongly Disagree to 6 = Strongly Agree. Statements centered on how values are aligned with one’s behaviors, such as “I act in ways that are consistent with how I wish to live my life.” Items were summed for a total score, with possible scores ranging from 0 to 48. Higher scores indicate greater engagement in valued behaviors. The measure demonstrated high internal consistency across all three timepoints (*α* = 0.81–0.86).

The Meaning Awareness Scale (MAS; Vagnini, 2020) was administered to assess meaning salience, or the extent to which participants were aware of and engaged with their sense of meaning in life in the moment. The 6-item scale included statements, such as “I was aware of the meaning in my life.” Participants were asked to reflect upon the past day and report how often they experienced that state, with 1 = Rarely to 7 = Very Often. Items were averaged for a total scale score, with higher scores indicating greater meaning salience. The MAS demonstrated high internal consistency across all three timepoints (α = 0.80–0.94).

The Snaith-Hamilton Pleasure Scale (SHAPS; Franken et al., 2007) was used to evaluate the capacity to experience pleasure from non-substance rewards. Participants considered how they would have felt in the last few days and indicate their level of agreement (0 = Definitely Agree, 1 = Agree, 2 = Disagree, 3 = Definitely Disagree) for the 14 statements, such as “I would enjoy being with family or close friends” and “I would find pleasure in my hobbies and pastimes.” Item responses are dichotomized (0,1), with item scores ≥2 recoded as 1, and then summed for a total score. Scores range from 0–14, with lower scores indicating greater pleasure derived from non-substance rewards. The SHAPS had high internal consistency across all three timepoints (*α* = 0.85–0.94).

#### 2.4.8 Engagement in treatment

Engagement in VBA was measured with three indices: completed VBA visits, length of VBA sessions, and goal completion. The BHC recorded the number of visits attended and the time duration of the sessions in minutes. Goal completion was measured using a validated observer coding system for BA sessions (Busch et al., 2010). During each session, the clinician recorded goals, reviewed their progress toward each goal (0 = Made no effort to begin; 1 = Made attempt/effort to start; 2 = Partial completion; 3 = Fully completed), and estimated the percent of each goal completed from 0–100%. For example, if a patient set a goal to go for a walk twice in the prior week, but did not go at all, completion would be 0%. If they went once, completion would be 50%. If they went twice, completion would be 100%.

### 2.5 Procedure

#### 2.5.1 Recruitment

Potentially eligible patients were mailed a letter that described the study procedures, risks and benefits, and compensation and invited them to participate. Interested individuals had the option to call the study team or wait for a phone call from the project coordinator. The project coordinator called individuals approximately one week after the letter was sent to assess patient interest in participating and screen for additional eligibility requirements. During the initial eligibility screening call, participants were asked whether they were still taking MOUD and were currently attending therapy at least monthly from a counselor or psychotherapist. Interested and eligible participants were scheduled for an in-person visit at the clinic to consent into the study, complete baseline measures, and hold their first VBA session.

#### 2.5.2 Enrollment

Individuals attended an in-person visit to provide informed consent and complete baseline measures. Participants initially met with the project coordinator and were given all required elements of consent, including a description of the study activities, potential risks and benefits of participation, the voluntary nature of the study, and who may have access to their data. They were given opportunities to ask questions before providing consent to participate. After signing the consent form, they were asked to complete baseline self-report measures and provide a urine specimen for urine toxicology screening.

#### 2.5.3 Data collection

Participants completed study measures at three timepoints: baseline, mid-treatment (6 weeks), and post-treatment (12 weeks). At each measurement visit, participants completed survey measures and provided a urine sample for urine toxicology. Survey responses were recorded in the HIPAA-compliant REDCap database (Research Electronic Data Capture).

#### 2.5.4 Intervention

After completing the consent and baseline visit, participants met with the counselor to complete their first session of VBA. Follow-up sessions were scheduled directly with the counselor and occurred over 12 weeks. Sessions were conducted in-person or via telehealth (i.e., telephone visit or videoconferencing), depending on patient preference. Sessions were audio recorded to ensure treatment fidelity, and the counselor was supervised by a licensed clinical psychologist (SAH) with consultation from another licensed psychologist and BA expert (AMB). Supervision occurred weekly, where the BHC reviewed each member of the current caseload, discussed progress and challenges, and reviewed pertinent sections of audio recordings to obtain feedback. Audio recordings were destroyed after they had been reviewed and discussed for supervision. Participants who did not want their sessions recorded had an option to decline and could still participate in the study.

#### 2.5.5 Qualitative interview

Upon conclusion of the study, a member of the research staff contacted participants to conduct a brief interview about patients’ experiences during the VBA study. Interviews were audio-recorded and transcribed. Participants were asked about their thoughts on the intervention and working with the therapist. Open-ended questions asked participants to report what they liked or did not like about the counseling sessions, or perhaps what they found to be most or least helpful and ideas of what to change or continue with the program. More specific questions explored patients’ opinions on the number of sessions, format (in-person v. telehealth), experiences working with the counselor, and location of the program and relationship with the clinic’s MOUD program. Study-related questions focused on the research procedures, such as the recruitment process, study survey measures, and compensation for participation.

#### 2.5.6 Compensation

Participants received incentives for completing study measures at an increasing rate to promote study retention and incentivize consistency: $30 for baseline, $40 for mid-treatment (6 weeks), and $50 for post-treatment (12 weeks). Additionally, for each VBA session, participants were compensated $5 to cover their use of phone minutes or data used on their electronic devices for virtual visits (telephone) or for transportation to in-person sessions. All study payments were made using ClinCard, a payment management system for clinical trials operated through Greenphire.

### 2.6 Analysis

Quantitative analyses were conducted in SAS 9.4. To assess feasibility, descriptive statistics were used to report recruitment percent (i.e., percent of those invited who agreed to participate), recruitment pace (i.e., the number of people who enrolled weekly and the number of weeks to recruit the desired sample size), and retention percent (i.e., the percent of enrolled patients who completed the counseling sessions).

To assess acceptability, we calculated the mean and standard deviation of the CSQ-8. An average CSQ-8 scale of 24 out of 32 was the pre-specified threshold for participant acceptability. Acceptability was also assessed using qualitative interviews. Qualitative interviews were coded using NVivo by two members of the study team (SAH, MLC). Framework matrix analysis was applied to identify themes and determine the acceptability of the intervention and the research process.

To assess the psychometric properties of the self-report measures used, we conducted descriptive statistics (including means, standard deviations, and ranges) on items and total scores. Internal consistency reliability at each time point was calculated using Cronbach’s *α*. In exploratory analyses, we examined changes in the measures over time using paired *t*-tests and Cohen’s *d* effect sizes, comparing change from baseline to posttreatment. Because this was a small, single arm pilot study, and the primary goal was not to detect differences within participants over time, the interpretation of these differences was based on the size of the observed effect rather than the statistical significance. However, using a two-tailed paired samples *t*-test, with 19 participants, 80% power, and *α* = 0.05, the study was powered to detect an effect size of Cohen’s *d* = 0.68 (a moderate to large effect).

## 3 Results

A total of 21 patients enrolled in VBA. The majority were female (67%) with an average age of 44.0 years (*SD* = 12.2). Most of the participants identified as non-Hispanic White (75%), with the remainder identifying as Black (5%), American Indian (10%), or multiple races (10%). Approximately 19% of participants were single, never married, whereas 43% were married and 38% were separated or divorced. More than half were employed full-time (57%), 14% were employed part-time, 19% were retired, and 10% were unemployed. One-third had a 2-year college degree or higher education. About half (49%) had an income of less than $40,000/year. Patients reported an average length of buprenorphine use of 60 months (5 years; *SD* = 45 months, range 10–216 months). At baseline, patients reported little substance use in the prior 30 days, with cannabis being the most frequently used (48%), followed by alcohol (19%), psychedelics (10%), benzodiazepines (5%) and opioids (5%). At baseline, four participants (19%) had a positive screen for depression on the PHQ-9. Generally, the group was very stable on buprenorphine, not depressed, and not using other substances.

### 3.1 Feasibility

To assess the feasibility of delivering VBA in a primary care setting, we monitored treatment recruitment and retention rates (see Figure 1). Letters were mailed to 108 patients, followed by recruitment calls by telephone. Of the 79 patients reached by phone, 4 (5%) were ineligible, 13 (17%) declined, 27 (34%) were lost to recruitment follow-up, and 35 (45%) expressed interest and scheduled a baseline visit. On average across the 31 weeks of study recruitment, 2 patients enrolled every three weeks.

Participants completed an average of 5.1 VBA sessions (*SD* = 1.6, range 1–6). Fourteen participants (67%) completed all 6 offered sessions. The first session averaged 48 min long (*SD* = 10 min) and follow-up sessions were 25 min long (SD = 8 min). Participants set an average of 12.9 activation goals (*SD* = 4.3) with an average goal completion rate of 78% (*SD* = 28%). Most participants (90%) completed 6-week surveys and 12-week surveys (90%). Both participants discontinued prior to the 6-week assessment: one participant died during the study (unrelated to the study) and another participant was lost to follow-up.

### 3.2 Acceptability

Treatment satisfaction was assessed quantitatively with the CSQ-8. Overall acceptability of the intervention was very high (*M* = 30.4, *SD* = 1.6). Of the individual items, 84% of participants rated the services as “excellent,” 89% would definitely recommend the program to a friend, and 95% would come back to the program if they needed help in the future.

#### 3.2.1 Qualitative feedback

Of the 21 participants enrolled in the study, 81% completed the post-intervention interview (*n* = 17). Qualitative themes are presented in Table 1. In general, participants found the program to be beneficial and recommended that the BA program continue to be offered to patients receiving MOUD.

**Table 1:** Qualitative feedback and themes from participants.

Theme	Supportive quote
What participants *liked* about the VBA program	
1. VBA program features: When asked what they liked about the program, participants commented mostly on goal setting, goal completion, accountability, and autonomy. Nearly half of those interviewed (*n* = 8) said they could not think of anything they did not like and would wish to change.	*I guess it made me focus more on the things that we talked about as far as goals go, you know? …It made me want to reach those goals whereas before I was like, yeah, whatever. Well, I never thought about it I guess… now it was like goals, and I wanted to meet them.* *I found [setting goals] very useful. A lot of times when you are going through your everyday life, you do not think that set goals look at how you are doing things on an everyday basis. And so, it kind of helped me to do that. And it is just something you kind of get out of the habit of doing, I guess.*
2. Working with the BHC in the VBA program: All patients said they enjoyed working with the BHC. Most interviewees (64%; *n* = 11) noted the BHC’s kindness, approachability, empathy, professionalism, caring manner, and support with goals.	*Sometimes I did not always do it, and she wasn’t like how my family would be. ‘What’s wrong with you,’ you know? ‘Why did not you,’ you know? She was real supportive through it and gave suggestions what I could do next time. It was helpful.* *[The BHC] gave me that structure to build off of so that I would know where I was going with it because it was new. I did not do goals or daily activities or anything of that nature. So, it opened me up in a good way. Like I said, not to have the overwhelming and to get to where I needed to go with that. But I definitely had full control over what I wanted to do.*
3. Supportive behavioral health in conjunction with MOUD care: Participants noted that having support was helpful for people on MOUD.	*I think it’s a great idea because I just think the medication itself unfortunately is not enough. If it was, then we would not have people on heroin still. So I just think you need other support services and everyone’s recovery is different. … we should have as much resources and different recovery options and support groups as possible.*
4. Structure and format of the intervention: Most participants (*n* = 12) felt the number of sessions in the program was just right for them. Participants were mixed on their preference for virtual v. in-person VBA sessions.	*I had a combination, and we did a phone, one phone interview. Everything else was in person. I prefer in person because it helps me to be able to see someone. Phone works I think for some things but maybe not in this case. It might be helpful but it’s just not, I do not like that means of communication. I spend too much of my life on the telephone when I was working. And I leave it at home when I can. So, I like in-person.*
5. Location of the program: Most participants (*n* = 13) said that having the VBA program offered at the clinic they come to for MOUD treatment was important to them. Only 2 participants said this was not important.	*To me it was critical. If the study would have said I had to go somewhere else, I probably would not have done it. Even if it was the same distance, I do not know. I’m a God dang kind of creature of habit, I get some things stuck in me. So it made it easier for me to come by having it here. I suppose I should not have said I would not have gone if it was somewhere else but it would have been harder*.
What participants *disliked* about the VBA program	
1. Challenges with setting goals: Some participants had difficulty identifying goals and would have preferred more guidance in choosing their goals.	*I guess the only thing maybe would’ve been like when I made the goals maybe getting more …helping me make them by coming up with things…maybe suggesting, oh, how about we do this to make this happen? I do not know if that is a thing if that’s what they do or if they want you to come up with it on your own, but like I said, it is giving me answers. And they do not know the answers so they cannot help me maybe in that sense, but it was almost like I felt like I do not know how to do it. There were a couple times in my mind where I was like, well, I do not know how to do that. Yeah, I have that goal, but I do not know how. How can I make it possible?*
Other participants felt some goals they set for themselves to be too surface level and wanted to be guided toward goals that addressed deeper concerns with potentially significant outcomes.	*Seeing it in that way or maybe if it was even set up that way like some longer-term goals that really would impact your life, but you never really thought about it or did not know how to begin how to get to that point. I mean I thought it was really cool that it is all focused on like enjoyment, but sometimes fulfillment is tackling things you need to get done too.*
2. Focus of the behavioral intervention: A few participants desired more time focused on supportive therapy to address mental health concerns.	*I do not know. I guess maybe that just not being able, like it wasn’t an actual like my own therapist I guess so we could not really dive deep into maybe issues I still have around anxiety of stuff like that.*
What *recommendations* participants shared for future VBA programming	
1. Continue the VBA program for patients receiving MOUD care: Everyone who completed the interview said the VBA program should continue to be offered to other patients receiving MOUD care at the clinic.	*Absolutely, yes… it was helpful, it was. And to have something from that to take away from that to use moving forward, something that really helps me, I would highly recommend that you guys keep that program going.* *I just really hope you put it as part of your guy’s system here because I really do believe it will help a lot of people get back on their feet.*
2. When to offer VBA to persons in recovery: Multiple participants thought the VBA program would be particularly beneficial to people earlier in recovery.	*I would tie it with the intake of the Suboxone patients … I do not know how I would have been at that time initially, but I think that would be at least a good time to introduce it. Because at that time for me, I was very open to anything that was going to get me off these opiates and not having withdrawal and not having terrible pain… And I knew what the Suboxone could do but [VBA] adds just such a nice other dimension to it to put the possibility out there that there’s reasons that this will work and reasons you can make it work and this is how you can do it.*
3. Further development of goals: Some participants would have preferred to build upon their goals or tailor the program for larger goals that were personally meaningful to them.	*I wonder for me if there would be a way to build on the goals I had set. It was sort of just like, okay, you have this goal. Do you want to keep doing that goal or do you want to switch to a different goal? Is there a way to kind of maximize that goal? But I mean [BHC] was awesome. It is nothing like with that. Maybe I wasn’t utilizing it how I should have been.*
4. Appointment frequency and length of program: Future offering of VBA programming may consider appointment frequency, duration of programming, and stage of recovery.	*Maybe for someone newer in recovery maybe they would need more hands on. Maybe every week but also maybe offer it every two weeks because that’s something people may not want to do it because they think, ‘Oh it’s a commitment every week,’ but you could offer. I did not need it every week, but I’m sure if I did, maybe [the BHC] would have been okay with that. I did not ask because I did not need that. But maybe that would be helpful for people who are newly in recovery because sometimes for people new in recovery, two weeks is almost like a long-term goal.*
6. Involvement of PCC: Participants were mixed as to whether PCCs should be involved with the VBA program. Most participants (*n* = 11) said “yes, have the PCC involved,” whereas one-third (*n* = 6) said “no, the PCC does not need to be involved.”	*Yeah. I think that [PCC] recognized that I was getting stuck by myself, and he cannot help me. He’s a busy guy. He cannot be doing this kind of stuff with me. So, yeah, I really, really, really like it, and I’m glad that to offer it to people that have had time to actually be sober to clear their - my brain is still clearing up. It’s been over a year.* *Maybe, I would just rather [PCC] not be… I do not know why. Just because yeah I just liked it personal.*

Part of the success of any behavioral health intervention is the rapport, trust, and relationship development between the patient and BHC. All participants who completed the interview reported they very much enjoyed working with the BHC. Multiple factors made the patient-BHC interactions highly beneficial, and study participants noted the BHC’s listening skills, accountability, kindness, and support with goal setting. Most participants found goal setting to be helpful in advancing their personal values-based plans. A few participants commented that they wanted more guidance with their goals or desired progression to deeper, meaningful goals. Other participants wanted less time spent on goal setting and more time for supportive therapy about life circumstances and mental health (i.e., anxiety). The flexibility of in-person or virtual visits was appreciated by participants, and the quality of the sessions were viewed as comparable across the two formats. Multiple participants shared that the BA program would have been beneficial to them earlier in their recovery and recommended offering the program to those just initiating care at the clinic but to not make this behavioral intervention mandatory.

### 3.3 Preliminary efficacy

#### 3.3.1 Intervention processes and psychological outcomes

Means and standard deviations at each assessment time point and changes in processes and psychological outcomes from baseline to 12 weeks are reported in Table 2. Specifically, there were small increases in activation, avoidance, and meaning salience from baseline to 12 weeks. There were minimal to no changes in valued directions or capacity to experience pleasure. There were small and moderate increases in life satisfaction and positive affect, respectively. However, there were no changes in negative affect or depression from baseline to 12 weeks.

**Table 2:** Changes in psychological processes and outcomes.

	Means			Baseline to 12-week comparison	
Variable	Baseline *N* = 21	6-week *n* = 19	12-week *n* = 19	Cohen’s *d*	*p*
Processes					
Activation	34.5 (7.7)	32.7 (9.6)	36.2 (9.2)	0.22	0.34
Meaning Salience	5.1 (1.5)	5.5 (1.2)	5.4 (1.2)	0.32	0.18
Valued Actions	38.7 (6.4)	38.9 (8.2)	39.0 (6.5)	0.09	0.71
Capacity to Experience Pleasure	1.1 (2.5)	1.7 (3.4)	1.2 (2.3)	−0.05	0.83
Outcomes					
Life Satisfaction	21.9 (6.7)	--	22.8 (7.5)	0.25	0.48
Depression^a^	7.4 (4.2)	7.6 (5.6)	7.7 (5.8)	0.09	0.83
Positive Affect	3.3 (0.8)	3.1 (0.7)	3.5 (0.6)	0.62	0.016
Negative Affect	2.1 (0.7)	2.1 (1.0)	2.1 (0.8)	−0.07	0.75

a Depression outcomes were measured with the CESD-10.

#### 3.3.2 Opioid abstinence

Abstinence from opioid use was assessed through U-Tox screening results collected at baseline, mid-treatment, and post-treatment. According to U-Tox results conducted at the clinic, 100% of patients were abstinent from opioids at baseline (*n* = 21), mid-treatment (*n* = 16), and post-treatment (*n* = 19). At mid-treatment, three participants had to complete their assessment virtually and did not provide a sample for U-Tox screening. Consistent with self-reported substance use, U-Tox results showed cannabis use, with some participant samples testing positive at baseline (42.9%), mid-treatment (43.8%), and post-treatment (36.8%). Participants were consistent with their MOUD, with buprenorphine detected in 100, 93.8, and 100% of samples at baseline, mid-treatment, and post-treatment. No illicit substances were detected in U-Tox samples.

## 4 Discussion

This open pilot trial demonstrated that VBA is a feasible and acceptable adjunctive treatment among patients receiving buprenorphine in primary care. Most participants finished a full course of VBA (~5 sessions over 12 weeks) and were retained for follow-up assessments. They rated the program as highly acceptable, with the participants appreciating the connection with the BHC, goal setting, and format flexibility of the sessions. Preliminary results indicate that the program may be associated with small to moderate increases in well-being (life satisfaction and positive affect); however, there were no changes in negative symptoms, likely due to the low prevalence of depression and negative affect at baseline. In addition, all patients remained abstinent to opioids during the treatment program, which could be reflective of the fact that participants had been on MOUD for an average of 5 years.

These results led to a new question: specifically, who needs adjunctive treatment to MOUD in primary care settings? The sample recruited for this pilot had been on MOUD for an average of 5 years. The majority (80%) were not experiencing depressive symptoms at baseline. Additionally, there was low comorbid substance use in this group, with cannabis being the most used comorbid substance at just under 50%. Our intention was not to recruit a sample that had been in treatment for a long time, as our inclusion criteria required only one month of buprenorphine use. However, there were no other inclusion criteria for symptoms (e.g., depression symptoms) nor was there an upper limit on the length of time participants could be on buprenorphine. Because participants had been in recovery for some time, there was little opportunity to improve upon their baseline negative symptoms. Perhaps in a group that had been on MOUD for a shorter amount of time differences in depressive symptoms would be more pronounced not only from improving underlying depression but also improving substance use recovery skills. Positive affect and life satisfaction did improve to a small to moderate effect, suggesting that in this highly stable group, there was still room to improve well-being. Interestingly, their capacity to experience pleasure did not increase; however, this group already had relatively low anhedonia. This finding suggests participants used their existing capacity to increase well-being. Prior research from both clinical and non-clinical settings showing BA interventions lead to a medium effect size improvement in well-being (Mazzucchelli et al., 2010); although our effects were in the same direction, they were smaller in size. Further, many participants reported benefit from participating in the program, yet they also stated in the qualitative interviews that the program might be most helpful for people earlier in treatment.

In addition to targeting patients earlier in their treatment trajectory, participants recommended some important improvements to the program. The goal of VBA is to encourage engagement in values-congruent, non-drug pleasurable activities to increase positive reinforcement in people’s lives. The treatment encouraged goals that (1) supported treatment goals; (2) were connected to values; and (3) were pleasurable. Participants had a range of experiences with goal setting prior to the treatment: some were very experienced goal setters whereas others had more difficulty identifying potential goals. Participants recommended tailoring goals more to their specific needs. Specifically, those who had more experience setting goals desired more in-depth coaching on how to make the goals more meaningful and connected to long-term goals. Experienced participants tended to feel the goals only scratched the surface of what could be possible. Those with less experience wanted more scaffolding and guidance in what goals to pick. They even wanted the BHC to recommend specific goals to set. Therefore, there needs to be some further adaptation of the treatment to patients’ needs.

Although not explicitly tested in this study, there may be inherent benefit of integrating values into BA. Completing a values exercise at the beginning of the intervention helps participants clarify their values and identify disconnects between their values and current behaviors. These gaps become prime targets for intervention, where participants can set goals that align with what is meaningful and valuable to them and engage in actions that are consistent to their values (Anshel, 2015). In addition to the benefit of aiding goal setting, incorporating values into an intervention may have added benefits of enhancing motivation (Kanter et al., 2010) and facilitating greater intentions to change (Landais et al., 2023) and positive emotions (Christie et al., 2017). Interestingly, valued actions did not increase over the course of the intervention. This suggests that further work could be done to enhance the values component of the intervention, such as emphasizing it more in the follow-up sessions and inquiring how well each goal matches a participant’s identified values.

This study is strengthened by building on prior work that has demonstrated that VBA is an efficacious treatment for mood and behavioral concerns (Dimidjian et al., 2011). The treatment was manualized to ensure fidelity. However, this pilot is limited by the small sample at a single clinic and lack of a comparison group. A larger study, with a randomized comparison group (e.g., a supportive therapy group not focused on goals or values), powered to detect clinically meaningful differences in substance use or well-being would address these concerns. Further, participants had been on long-term treatment for OUD with buprenorphine. The feasibility and acceptability of the treatment in patients just starting treatment (< 6 months duration) is unknown. Recruiting participants who have recently started MOUD treatment (e.g., within 1–2 months) would help determine if the treatment is helpful for those just starting treatment.

In summary, VBA adapted for patients with OUD seems to be a feasible and acceptable adjunctive treatment for patients receiving MOUD in primary care settings. Future research using larger samples with comparison groups is needed to determine the efficacy of the treatment in improving substance use abstinence and well-being above and beyond MOUD.

## Data Availability

The raw data supporting the conclusions of this article will be made available by the authors, without undue reservation.
